# Kynurenine facilitates renal cell carcinoma progression by suppressing M2 macrophage pyroptosis through inhibition of CASP1 cleavage

**DOI:** 10.1515/biol-2025-1174

**Published:** 2025-10-08

**Authors:** Wenmao Huang, Jingxuan Chen

**Affiliations:** Department of Urology, 900th Hospital of PLA Joint Logistic Support Force, No. 156, North West Second Ring Road, Gulou District, Fuzhou, Fujian, 350000, China; Department of Cardiothoracic Surgery, 900th Hospital of PLA Joint Logistic Support Force, Fuzhou, China

**Keywords:** kynurenine, renal cell carcinoma, M2 macrophage, CASP1, tumor microenvironment

## Abstract

Renal cell carcinoma (RCC) is an aggressive malignancy with a poor prognosis influenced by pyroptosis in tumor-associated M2 macrophages. This study investigated how kynurenine modulates pyroptosis in M2 macrophages and promotes RCC progression. M2 macrophages were treated with pyroptosis inhibitor VX-765 or kynurenine to evaluate their effects on cell viability and pyroptosis. Transwell co-culture systems were employed to assess the impact of M2 macrophages on RCC cell proliferation, colony formation, and viability. The interaction between kynurenine and CASP1 (caspase-1), a key executor of pyroptosis that cleaves gasdermin D (GSDMD) to trigger inflammatory cell death, was analyzed using surface plasmon resonance. The results demonstrated that VX-765 treatment significantly enhanced M2 macrophage viability while reducing pyroptosis, thereby promoting RCC cell proliferation in co-culture systems. Kynurenine significantly enhanced M2 macrophage viability while suppressing pyroptosis. Mechanistically, kynurenine reduced the cleavage of CASP1 (caspase-1) by directly binding to it. Overexpression of CASP1 reversed kynurenine-induced suppression of pyroptosis in M2 macrophages. Furthermore, CASP1 overexpression abolished kynurenine-mediated enhancement of RCC cell viability, colony formation, and proliferation. This study revealed that kynurenine inhibits pyroptosis in M2 macrophages via direct targeting of CASP1, creating a tumor-supportive microenvironment that accelerates RCC progression. These findings establish the kynurenine–CASP1 axis as a critical regulator of M2 macrophage pyroptosis and demonstrate its role in promoting RCC progression, identifying a potential therapeutic target for RCC treatment.

## Graphical abstract

**Figure:**
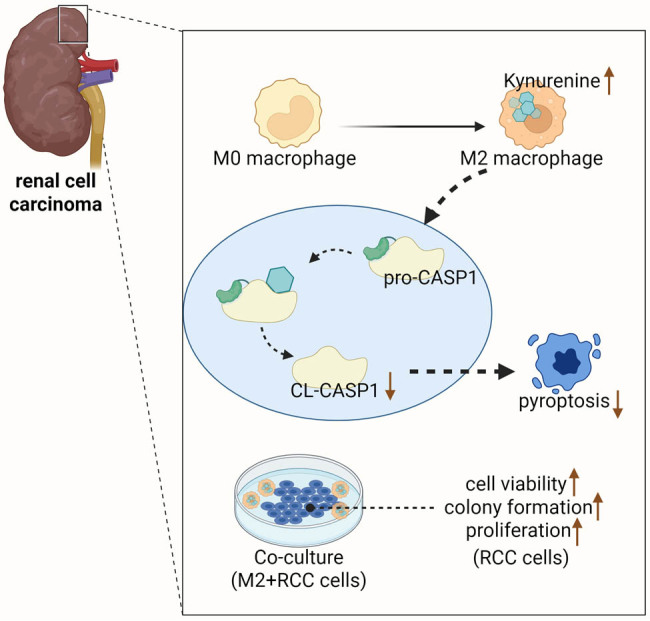

## 1 Introduction

Renal cell carcinoma (RCC) is one of the most common malignancies of the urinary system, accounting for approximately 2–3% of all adult cancers [1]. The incidence of RCC has shown a steady increase over the past few decades, with an estimated 400,000 new cases diagnosed globally each year [2]. Despite significant advancements in surgical techniques and targeted therapies, the prognosis for advanced RCC remains poor, with a 5-year survival rate below 10% for metastatic disease [3]. These statistics highlight the critical need to unravel the molecular mechanisms driving RCC progression and identify novel therapeutic targets.

The tumor microenvironment (TME) plays a central role in cancer progression, with macrophages representing one of the most abundant immune cell populations within this milieu [4]. Macrophages can be polarized into two distinct phenotypes: classically activated M1 macrophages that exhibit anti-tumor properties, and alternatively activated M2 macrophages that promote tumor growth, angiogenesis, and immune suppression [5]. In the context of RCC, M2 macrophages are particularly prevalent and strongly correlated with adverse clinical outcomes [6,7]. Pyroptosis, a form of programmed cell death characterized by cellular swelling, membrane rupture, and release of pro-inflammatory cytokines, has gained increasing recognition as a critical process in cancer biology [8,9]. However, the specific role of pyroptosis in M2 macrophages and its implications for RCC progression remain poorly characterized.

Kynurenine serves as a critical metabolite in the tryptophan metabolic pathway. Tryptophan undergoes catabolism via the kynurenine pathway (KP) to generate diverse bioactive compounds, with kynurenine functioning as a key intermediate [10]. This metabolic route plays a pivotal role in immune regulation [11], neurological function [12], and oncogenesis [13]. Elevated kynurenine levels have been detected in multiple cancers [14], including RCC, and are associated with immune evasion and tumor progression [15]. Recent evidence suggests that kynurenine, a key immunosuppressive metabolite in the TME, can modulate macrophage function by influencing inflammatory signaling pathways [16,17]. Given that pyroptosis is primarily regulated by caspase-1 (CASP1) and that kynurenine has been implicated in inflammatory regulation, we hypothesized that kynurenine might suppress pyroptosis in M2 macrophages by targeting CASP1, thereby promoting RCC progression. However, this potential interaction and its functional consequences remain unexplored.

This study systematically examines the role of kynurenine in regulating pyroptosis in M2 macrophages and its impact on RCC progression. Our findings elucidate the molecular mechanisms underlying kynurenine-mediated modulation of macrophage behavior in RCC, providing novel insights into tumor-immune interactions.

## 2 Methods

### 2.1 Cell culture and treatment

M0 macrophages and RCC cell lines (786-O and A498) were obtained from ACTT (Manassas, VA, USA). Cells were maintained in RPMI-1640 medium supplemented with 10% fetal bovine serum and 1% penicillin–streptomycin under standard culture conditions (37°C, 5% CO_2_). All reagents were sourced from ATCC. M0 macrophages were seeded at a density of 1 × 10^6^ cells/mL in culture plates. M2 polarization was induced by treatment with interleukin-4 (IL-4) and interleukin-13 (IL-13) (20 ng/mL each) for 72 h. M2 macrophages were pre-treated with 50 μM pyroptosis inhibitor VX-765 (MedChemExpress, Monmouth Junction, NJ, USA), 10 μM ferroptosis inhibitor Fer-1, or 20 μM apoptosis inhibitor Z-VAD-FMK for 4 h. M2 macrophages were exposed to kynurenine at concentrations of 0.25, 0.5, 1, or 2 mmol/L for 48 h.

To assess tumor cell behavior in co-culture with M2 macrophages, Transwell inserts with 0.4 µm pore size were utilized. RCC cells were seeded in the lower chamber of a 24-well Transwell system and allowed to adhere for 4 h. After adherence, M2 macrophages were evenly distributed onto the upper membrane of the Transwell insert. The macrophage suspension was carefully added to the upper chamber to ensure uniform coverage. The co-culture system was then incubated at 37°C in 5% CO_2_ for 48 h to evaluate intercellular interactions.

### 2.2 Cell transfection

CASP1-overexpressing plasmids and control empty vectors were obtained from Shanghai GenePharma. M2 macrophages were transfected with the designated plasmids using Lipofectamine 3000 reagent (Invitrogen, Carlsbad, CA, USA) according to the manufacturer’s protocol.

### 2.3 Quantitative real-time PCR (qPCR)

Total RNA was isolated from samples following a standardized protocol. Subsequently, 1 μg of purified RNA was reverse-transcribed to cDNA using a commercial reverse transcription kit (Takara, Tokyo, Japan). The resulting cDNA was analyzed via quantitative real-time PCR on the LightCycler 480 system (Roche, Basel, Switzerland). The reaction mixture comprised SYBR Green Master Mix (Takara) as the fluorescent detection reagent. Relative gene expression levels were quantified using the comparative CT (2^−∆∆CT^) method, with normalization to housekeeping genes.

### 2.4 Cell viability assay

Cell viability was assessed using the Cell Counting Kit-8 (CCK-8; Beyotime, Shanghai, China). Briefly, cells were incubated with 10 μL CCK-8 reagent at 37°C in a humidified atmosphere for 2 h. The optical density at 450 nm was measured using a microplate reader (BMG Labtech, Offenburg, Germany) to quantify cell viability.

### 2.5 Pyroptosis detection by flow cytometry

Cellular pyroptosis was quantified using the FAM-FLICA Caspase-1 Assay Kit (ImmunoChemistry Technologies, Bloomington, MN, USA). Following experimental treatments, cells were harvested and washed with phosphate-buffered saline. The cell pellets were resuspended in wash buffer and co-stained with FLICA reagent and propidium iodide (PI) for 30 min at room temperature in the dark. Fluorescence signals were analyzed using a FACS Calibur flow cytometer (BD Biosciences, San Jose, CA, USA). The percentage of pyroptotic cells was calculated based on the dual fluorescence intensity of FLICA and PI.

### 2.6 Western blot analysis

Total protein was extracted from cells or tissues using a lysis buffer. Protein concentrations were determined using a BCA protein assay kit (Abcam, Cambridge, MA, USA). Equal quantities of protein (50 µg per lane) were resolved via electrophoresis on 10% sodium dodecyl sulfate-polyacrylamide gel electrophoresis gels and transferred onto polyvinylidene fluoride membranes. Membranes were blocked with a rapid protein-blocking solution to minimize nonspecific binding and incubated overnight at 4°C with primary antibodies diluted at 1:1,000. The following primary antibodies were employed: anti-pro-CASP1 (ab179515, Abcam), anti-cleaved-CASP1 (#89332, Cell Signaling Technology, Danvers, MA, USA), and anti-GAPDH (ab8245, Abcam) as a loading control. After washing, membranes were incubated with horseradish peroxidase-conjugated secondary antibodies (Abcam) for 2 h at room temperature. Protein bands were visualized using a ChemiDoc™ XRSC imaging system (Bio-Rad, Hercules, CA, USA), and band intensities were quantified using Image Lab software.

### 2.7 EdU incorporation assay

Cell proliferation was evaluated using the 5-ethynyl-2′-deoxyuridine (EdU) incorporation assay (Ribobio, Guangzhou, China). RCC cells were seeded in 24-well plates at a density of 2 × 10^4^ cells/well and allowed to adhere under standard culture conditions. Following incubation with 50 µmol/L EdU reagent for 2 h, cells were washed with PBS and fixed in 4% paraformaldehyde for 15 min at room temperature. Permeabilization was performed using 0.5% Triton X-100 (Beyotime, Shanghai, China) for 10 min, followed by nuclear counterstaining with DAPI. EdU-positive cells were visualized using a confocal microscope (Olympus, Tokyo, Japan), and proliferation rates were quantified by calculating the ratio of EdU-positive cells to total DAPI-stained nuclei.

### 2.8 Colony formation assay

The clonogenic potential of RCC cells was assessed using a colony formation assay. Cells were seeded in 6-well plates at a density of 1 × 10³ cells/well and cultured under standard conditions (37°C, 5% CO_2_) for 14 days. Following incubation, cells were fixed with 4% paraformaldehyde for 15 min and subsequently stained with 0.2% crystal violet solution for 15 min. Excess dye was removed by rinsing with distilled water, and plates were air-dried completely. Colonies containing ≥50 cells were manually counted under an inverted microscope (Olympus, Tokyo, Japan). Clonogenic efficiency was calculated as the percentage of seeded cells that formed colonies relative to the total number of seeded cells.

### 2.9 Liquid chromatography-tandem mass spectrometry (LC-MS/MS)

Kynurenine was quantified by LC-MS/MS. Supernatant samples were mixed with 80% methanol and evaporated to dryness. Cellular extracts were prepared by homogenizing pellets in 80% methanol at −80°C, followed by centrifugation. Chromatographic separation was achieved on an ACQUITY UPLC BEH Amide Column (2.1 mm × 100 mm, 1.7 μm) using a binary gradient. Mobile phase A consisted of 0.1% formic acid in acetonitrile, and mobile phase B contained 10 mM ammonium formate with 0.1% formic acid. The gradient was 20% B (3 min), linear ramp to 50% B (12 min), hold (15 min), and return to 20% B (18 min). Detection used positive ESI on an API 4000™ system with 5 µL injection. Data were normalized to cell count.

### 2.10 Surface plasmon resonance (SPR) technology

Recombinant CASP1 protein was diluted in sodium acetate buffer (pH 5.0) and immobilized onto a sensor chip via amine coupling chemistry. Post-immobilization, residual reactive groups on the chip surface were blocked with 1 M ethanolamine hydrochloride (pH 8.5) to minimize non-specific binding. Kynurenine was serially diluted in running buffer (10 mM HEPES, 150 mM NaCl, 0.005% Tween 20, pH 7.4) to generate a concentration gradient (0.125–2 μM). The diluted kynurenine solutions were injected over the CASP1-immobilized sensor chip at a flow rate of 30 µL/min. Binding interactions were monitored in real time using an SPR biosensor system. Following each binding cycle, the chip surface was regenerated with a mild solution of 10 mM glycine-HCl (pH 2.0) to remove bound kynurenine without compromising immobilized CASP1 integrity. SPR sensorgrams were analyzed using the manufacturer’s software to subtract baseline signals and corrected for refractive index drift. Binding kinetics, including association rate constants (*k*_a_), dissociation rate constants (*k*_d_), and equilibrium dissociation constants (*K*_D_), were calculated by global fitting of sensorgram data to a 1:1 Langmuir binding model. The affinity of kynurenine–CASP1 interaction was quantified based on these kinetic parameters.

### 2.11 Statistical analysis

Data are presented as mean ± standard deviation (SD) from ≥3 biologically independent experiments. All statistical analyses were performed using GraphPad Prism 8.3 (GraphPad Software, San Diego, CA, USA). Two-group comparisons were conducted using Student’s *t*-test, while multiple-group comparisons were analyzed via one-way ANOVA with Tukey’s *post hoc* test. A *P*-value of <0.05 was considered statistically significant.

## 3 Results

### 3.1 VX-765 effectively inhibits the pyroptosis of M2 macrophages

**Figure 1:**
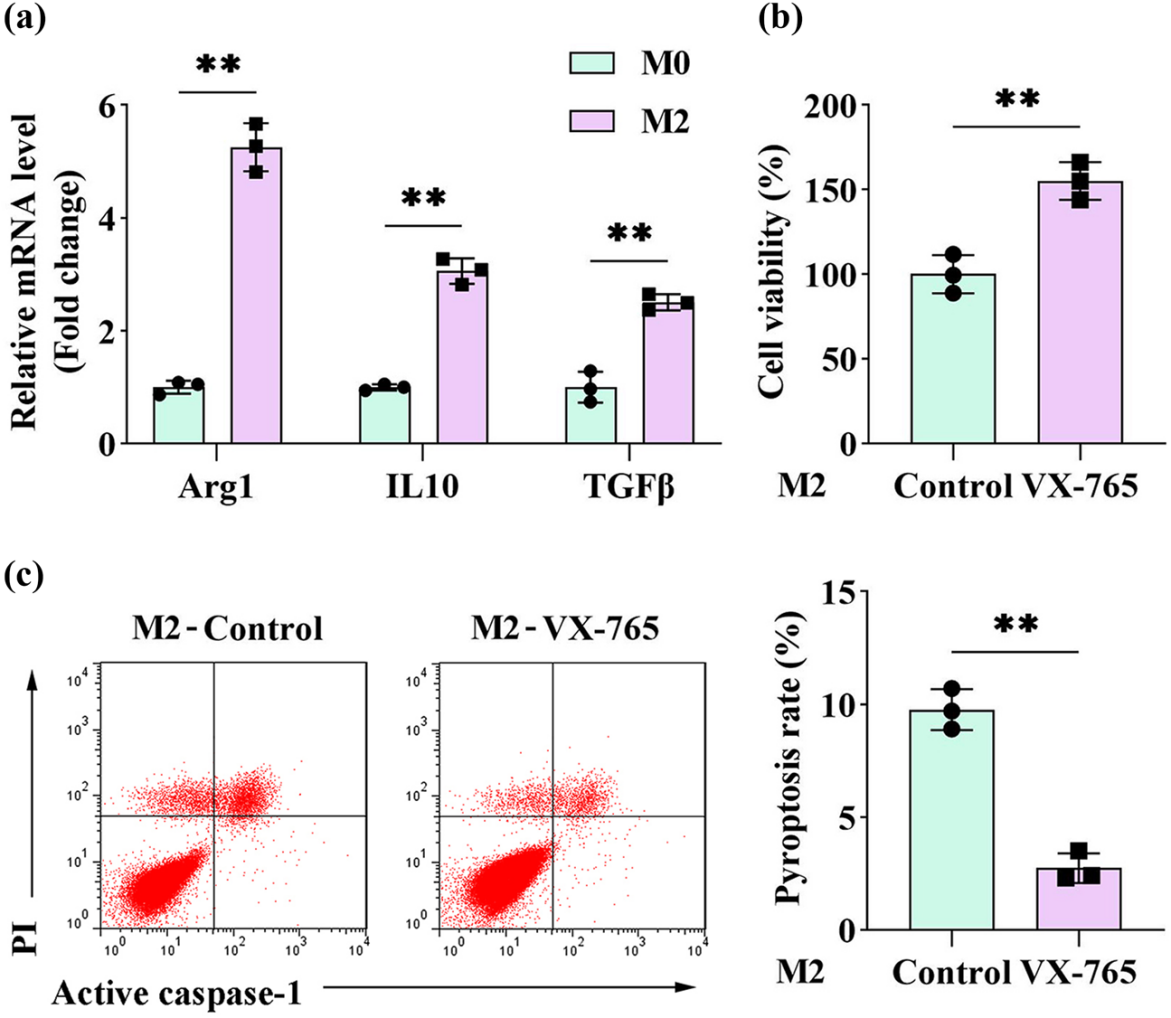
VX-765 effectively inhibited the pyroptosis of M2 macrophages. (a) Following polarization with IL-4 and IL-13, M2 macrophages were assessed for characteristic markers (Arg1, IL-10, and TGF-β) using qPCR. (b) and (c) M2 macrophages were treated with pyroptosis inhibitors (VX-765). (b) Cell viability was measured by the CCK-8 assay. (c) The pyroptosis rate was measured by flow cytometry. (*n* = 3) All data are expressed as the mean ± SD. **P < 0.01.

Upon stimulation with IL-4 and IL-13, M0 macrophages were polarized into the M2 phenotype. qPCR analysis demonstrated significant upregulation of M2 macrophage markers, including arginase-1 (Arg1), interleukin-10 (IL-10), and transforming growth factor-beta (TGF-β), in these polarized cells (Figure 1a). Exposure of M2 macrophages to the pyroptosis inhibitor VX-765 significantly increased cell viability while markedly reducing the pyroptosis rate (Figure 1b and c).

### 3.2 Inhibition of pyroptosis in M2 macrophages promotes the proliferation of RCC cells in the co-culture system

**Figure 2:**
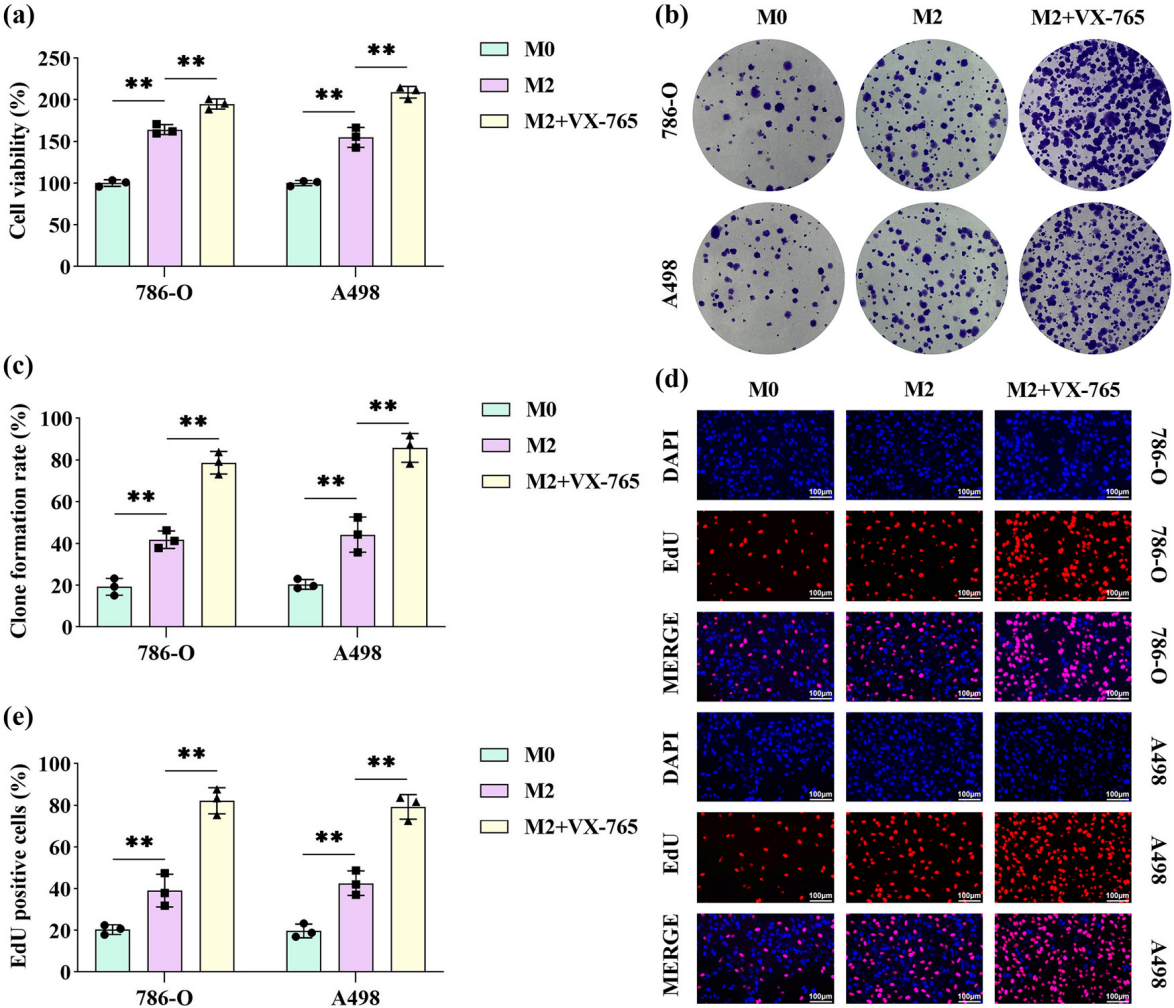
Inhibition of pyroptosis in M2 macrophages promoted the proliferation of RCC cells in the co-culture system. M2 macrophages, either treated or untreated with VX-765, were co-cultured with RCC cells using a Transwell system. (a) RCC cell viability was measured by the CCK-8 assay. (b) and (c) The proliferative capacity of RCC cells was assessed using a colony formation assay. (d) and (e) The proliferative ability of RCC cells was evaluated using an EDU proliferation assay; scale bar: 100 μm (*n* = 3). All data are expressed as the mean ± SD. **P < 0.01.

To investigate the effects of M2 macrophages on RCC cells, M2 macrophages were treated with or without VX-765 and subsequently co-cultured with RCC cells in a Transwell system. Cell viability analysis revealed that RCC cells in the M2 macrophage co-culture system exhibited significantly higher viability compared to those co-cultured with M0 macrophages. This viability enhancement was further amplified following VX-765 treatment (Figure 2a). Colony formation assays demonstrated that M2 macrophages significantly promoted RCC cell proliferation, and this effect was markedly augmented when pyroptosis in M2 macrophages was inhibited (Figure 2b and c). Consistently, EdU incorporation assays corroborated these findings, showing increased EdU-positive cells in the M2 macrophage co-culture system, particularly under VX-765 treatment (Figure 2d and e).

### 3.3 Kynurenine inhibits the pyroptosis of M2 macrophages in a dose-dependent manner

**Figure 3:**
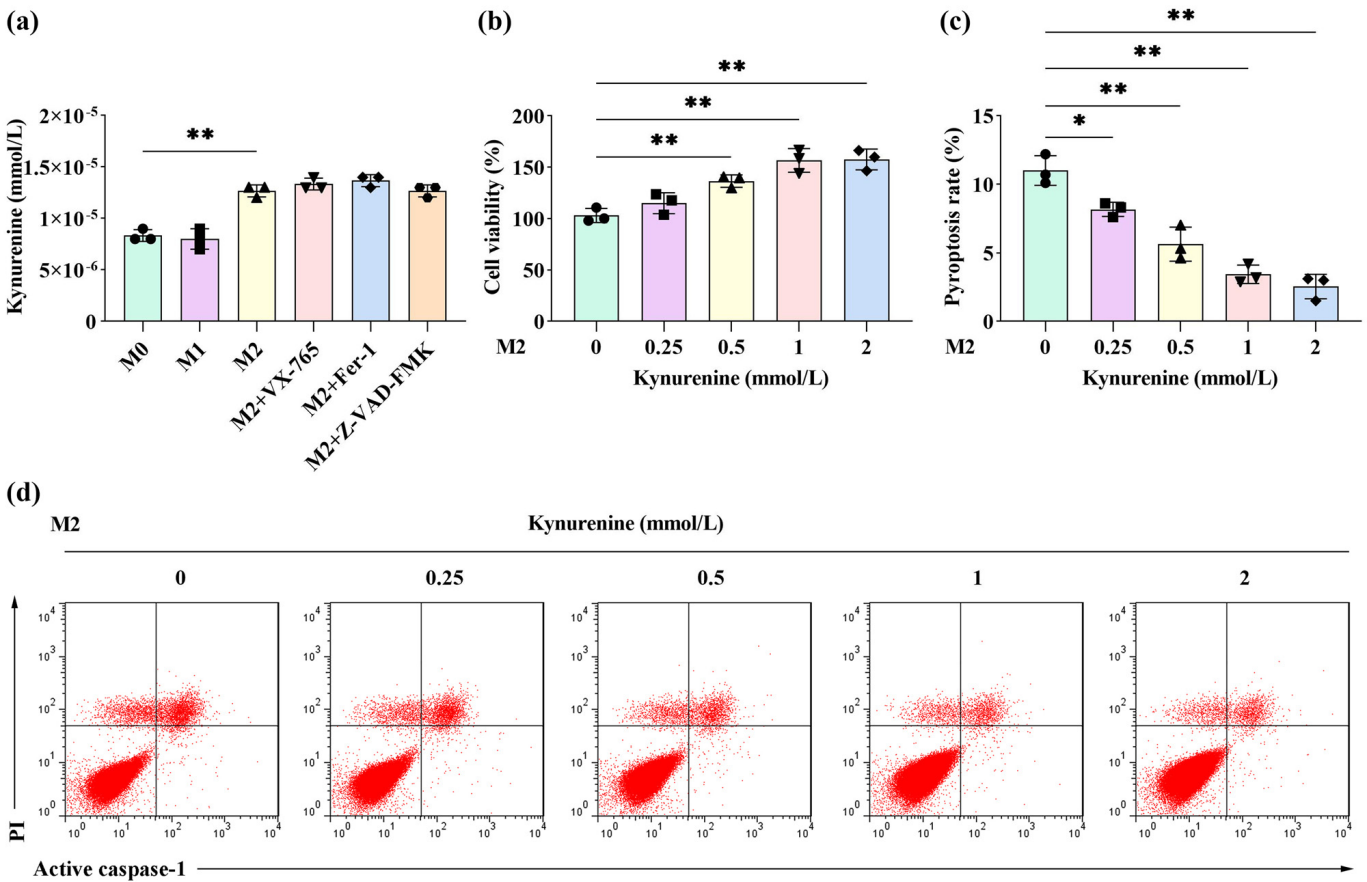
Kynurenine inhibited the pyroptosis of M2 macrophages in a dose-dependent manner. (a) The level of kynurenine in M0 macrophages, M1 macrophages, M2 macrophages, and M2 macrophages using different inhibitors (VX-765, Fer-1, Z-VAD-FMK) was measured via LC/MS/MS. (b)–(d) After M2 macrophages were treated with 0.25, 0.5, 1, and 2 mmol/L kynurenine, (b) cell viability was measured by the CCK8 assay. (c) and (d) The pyroptosis rate was measured by flow cytometry (*n* = 3). All data are expressed as the mean ± SD. *P < 0.05 and **P < 0.01.

Compared to M0 macrophages, kynurenine levels were significantly elevated in M2 macrophages. Measurement of kynurenine concentrations in M2 macrophages treated with various inhibitors revealed no significant alterations in its levels (Figure 3a). Subsequent analysis of kynurenine’s effects on M2 macrophage viability demonstrated that exposure to varying concentrations of kynurenine significantly enhanced cell viability in a dose-dependent manner (Figure 3b). Concurrently, kynurenine progressively suppressed the pyroptosis rate of M2 macrophages in a concentration-dependent fashion (Figure 3c and d).

### 3.4 Kynurenine decreases its levels in M2 macrophages by interacting with CASP1

**Figure 4:**
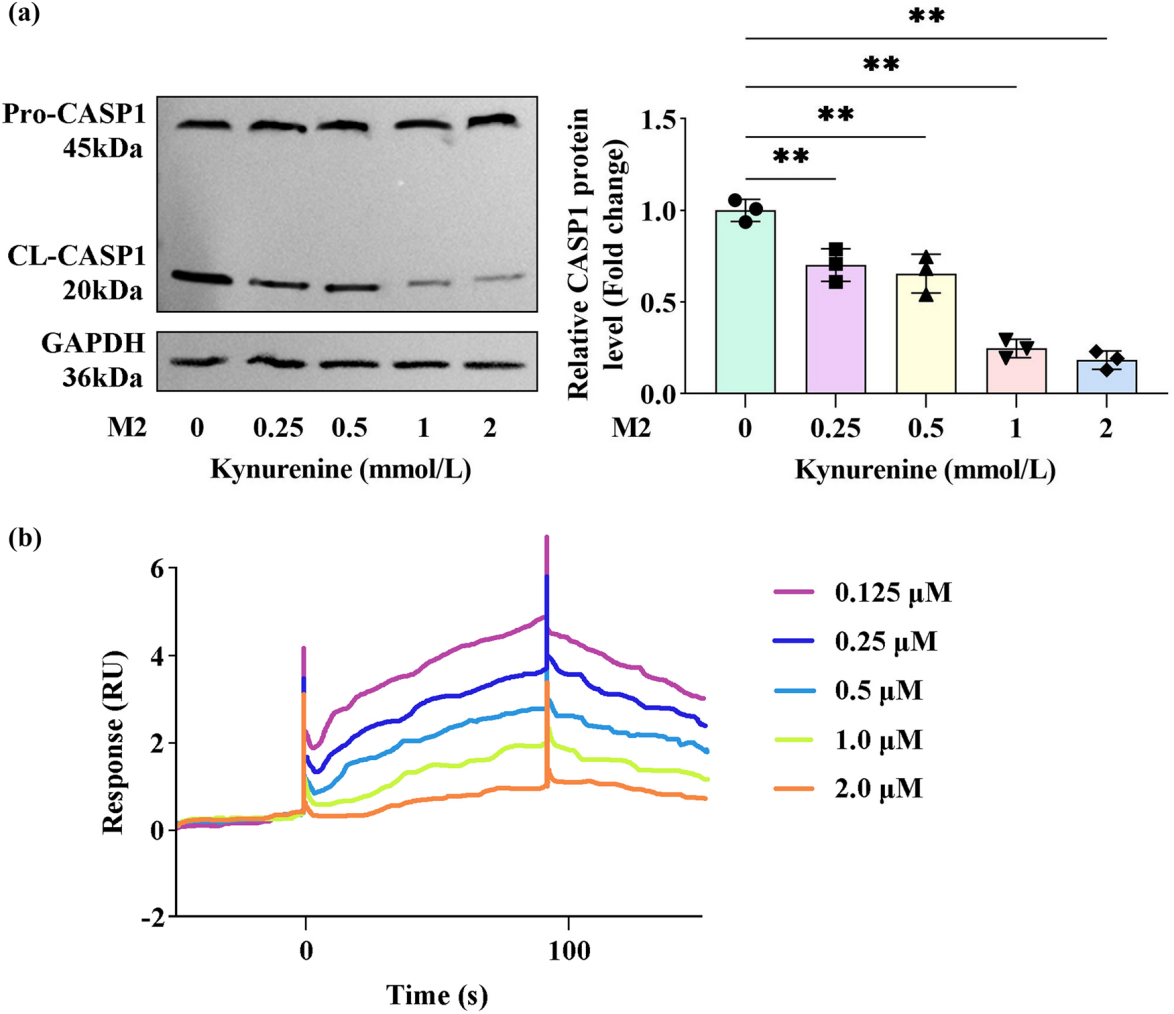
Kynurenine decreased its levels in M2 macrophages by interacting with CASP1. After M2 macrophages were treated with 0.25, 0.5, 1, and 2 mmol/L kynurenine, (a) the expression of pro-CASP1 and CL-CASP1 was measured by western blotting. (b) The interaction between kynurenine and CASP1 was confirmed using SPR technology. (*n* = 3) All data are expressed as the mean ± SD. **P < 0.01.

To investigate the molecular mechanism underlying kynurenine’s anti-pyroptotic effects, we performed western blot analysis to quantify both pro-CASP1 and cleaved CASP1 (CL-CASP1) in M2 macrophages treated with escalating kynurenine concentrations. As shown in Figure 4a, CL-CASP1 levels in M2 macrophages exhibited a dose-dependent decline with increasing kynurenine treatment. To further validate this interaction, SPR technology was employed to confirm direct binding between kynurenine and CASP1, demonstrating a measurable binding affinity (Figure 4b).

### 3.5 Overexpression of CASP1 reverses the inhibitory effect of kynurenine on pyroptosis in M2 macrophages

**Figure 5:**
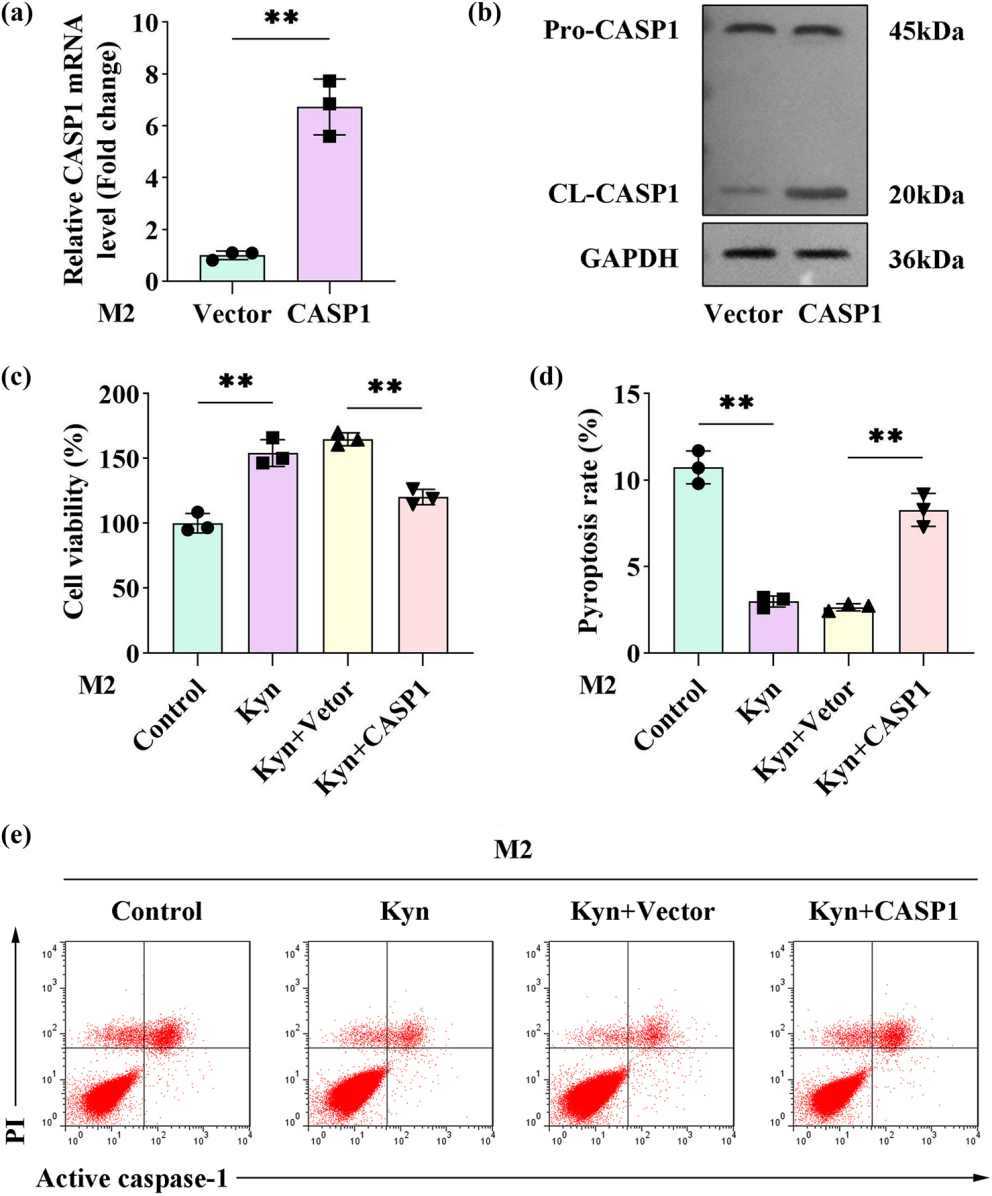
Overexpression of CASP1 reversed the inhibitory effect of kynurenine on pyroptosis in M2 macrophages. (a) After transfecting M2 macrophages with the CASP1 overexpression plasmid, the mRNA level of CASP1 was detected by qPCR. (b) The expression of pro-CASP1 and CL-CASP1 was measured by western blotting. (c)–(e) M2 macrophages were treated with kynurenine and transfected with or without the CASP1 overexpression plasmid. (c) Cell viability was measured by the CCK8 assay. (d) and (e) The pyroptosis rate was measured by flow cytometry. (*n* = 3) All data are expressed as the mean ± SD. **P < 0.01.

To elucidate the functional role of CASP1, M2 macrophages were transfected with a CASP1 overexpression plasmid, resulting in a significant upregulation of CASP1 expression (Figure 5a and b). CCK-8 assays demonstrated that the kynurenine-induced enhancement of M2 macrophage viability was attenuated by CASP1 overexpression (Figure 5c). Furthermore, the kynurenine-mediated suppression of pyroptosis in M2 macrophages was reversed by CASP1 overexpression, as evidenced by reduced pyroptosis rates (Figure 5d and e).

### 3.6 Overexpression of CASP1 reverses the induction of kynurenine on the malignant behavior of RCC cells

**Figure 6:**
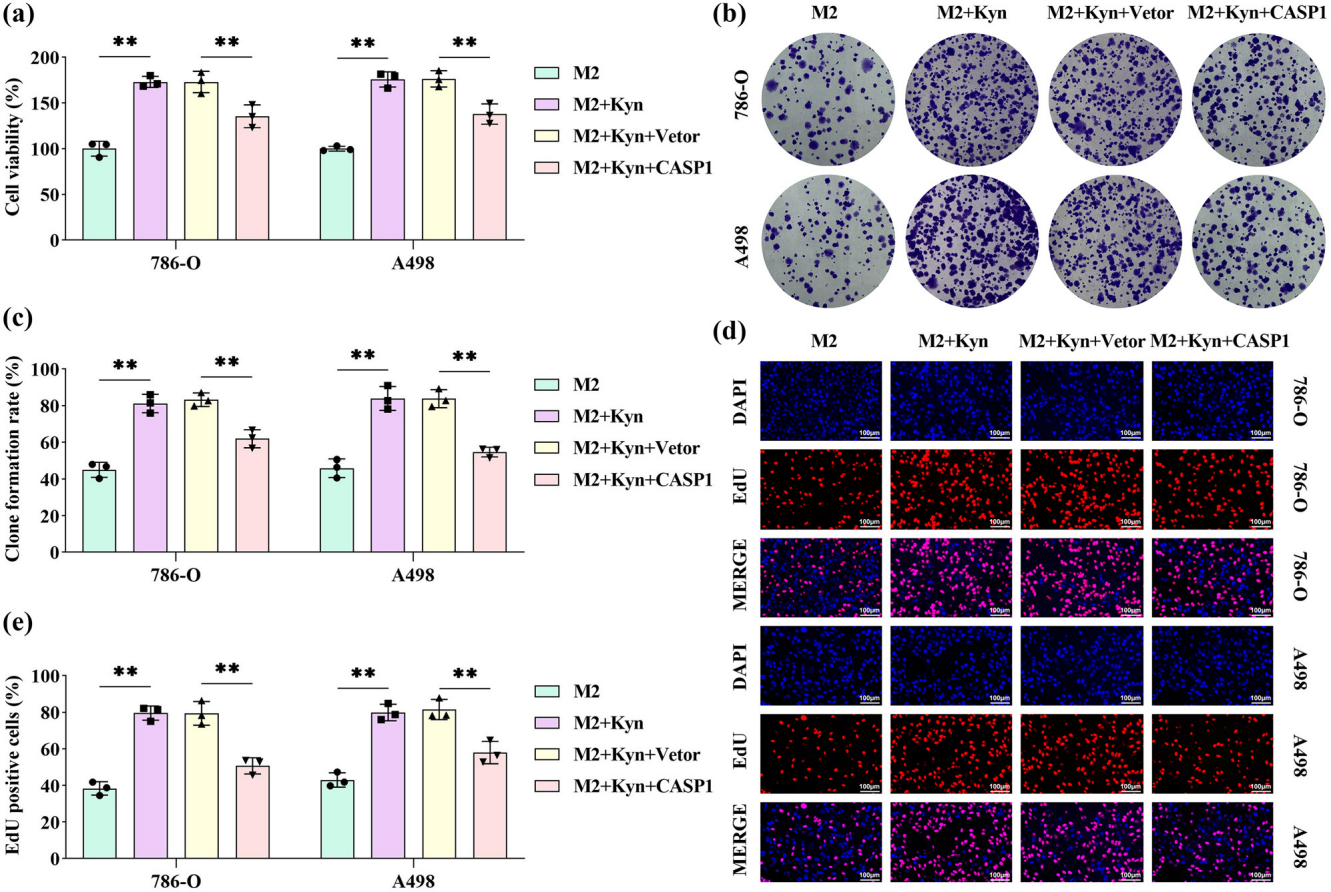
Overexpression of CASP1 reversed the induction of kynurenine on the malignant behavior of RCC cells. M2 macrophages were treated with kynurenine, transfected with CASP1 overexpression plasmid, and co-cultured with RCC cells. (a) RCC cell viability was measured by the CCK-8 assay. (b) and (c) The proliferative capacity of RCC cells was assessed using a colony formation assay. (d) and (e) The proliferative ability of RCC cells was evaluated using an EDU proliferation assay; scale bar: 100 μm (*n* = 3). All data are expressed as the mean ± SD. **P < 0.01.

In the co-culture system of M2 macrophages and RCC cells, kynurenine significantly enhanced cell viability (Figure 6a), colony-forming capacity (Figure 6b and c), and proliferative potential (Figure 6d and e) of RCC cells. However, these kynurenine-induced effects were abrogated by CASP1 overexpression, as evidenced by restored cell viability, reduced colony formation, and suppressed proliferation (Figure 6a–e).

## 4 Discussion

RCC is a cancer characterized by an immunosuppressive TME and well-known metabolic background, where tumor-associated macrophages (TAMs), particularly the M2 phenotype, play a crucial role in promoting tumor progression. Pyroptosis regulates macrophage function and immune responses, while the tryptophan metabolite kynurenine has emerged as a key immunomodulator in cancer. Our initial experiments demonstrated that treatment with the pyroptosis inhibitor VX-765 significantly enhanced M2 macrophage viability while reducing their pyroptosis rate. This finding highlights the critical role of pyroptosis as a regulatory mechanism governing macrophage survival and function [18]. Pyroptosis is typically associated with the release of pro-inflammatory cytokines and the activation of anti-tumor immune responses [8]. However, in the context of M2 macrophages, which are known to facilitate tumor growth and immune evasion, the inhibition of pyroptosis appears to amplify their pro-tumorigenic functions [19]. This observation aligns with previous studies demonstrating that pyroptosis can exhibit dual roles in cancer, either promoting or suppressing tumor progression depending on the cellular context [20]. For instance, in certain cancers, pyroptosis of tumor cells triggers anti-tumor immunity, whereas pyroptosis of immune cells can induce an immunosuppressive microenvironment [21]. Our findings indicate that in RCC, the suppression of pyroptosis in M2 macrophages promotes tumor progression.

Co-culture experiments revealed that M2 macrophages significantly enhance the proliferation and malignant behavior of RCC cells, and this effect is further amplified when pyroptosis is inhibited. This observation highlights the critical role of M2 macrophages in modulating the TME to facilitate cancer progression. M2 macrophages are known to secrete pro-angiogenic factors, cytokines, and extracellular matrix components that drive tumor angiogenesis, invasion, and immune suppression. For instance, M2 macrophages have been shown to secrete growth factors such as vascular endothelial growth factor and TGF-β, which directly promote tumor growth and metastasis [22]. By inhibiting pyroptosis, these macrophages may become more resistant to programmed cell death, enabling them to persist in the TME and amplify their pro-tumorigenic activities [23]. Conversely, inducing pyroptosis and reprogramming TAMs could enhance anti-tumor immune responses [24]. For example, dysregulation of key enzymes in TAMs has been linked to pyroptotic cell death, which may paradoxically promote immune evasion in colorectal cancer [25].

Tryptophan is enzymatically converted by indoleamine 2,3-dioxygenase (IDO) or tryptophan 2,3-dioxygenase (TDO) to form *N*-formylkynurenine, which is subsequently metabolized to kynurenine. This KP metabolite plays key roles in immunomodulation, neuromodulation, and redox homeostasis [26]. Accumulating evidence indicates that IDO and kynurenine mediate immunosuppression primarily through three mechanisms: inhibiting T-cell effector function, promoting regulatory T-cell (Treg) activation, and suppressing natural killer cell cytotoxicity [27]. The KP maintains a delicate balance between neuroprotective and neurodegenerative processes in the central nervous system, with its dysregulation contributing to the pathogenesis of Parkinson’s disease [28]. Notably, KP enzymes and metabolites exhibit redox sensitivity, functioning as either reactive oxygen species scavengers or generators depending on cellular metabolic states [29]. Our findings revealed that kynurenine significantly enhances M2 macrophage viability and suppresses pyroptosis in a dose-dependent manner. This observation is particularly noteworthy given kynurenine’s well-established role as an immune modulator. By suppressing T-cell activity and amplifying the function of immunosuppressive cells – including regulatory Tregs and myeloid-derived suppressor cells – kynurenine promotes immune tolerance [30,31]. In cancer biology, KP-mediated autophagy in cervical carcinoma cells has been shown to enhance macrophage phagocytic activity [32], while targeting tryptophan catabolism in ovarian cancer attenuates TAM infiltration and subsequent tumor progression [33]. Our study further demonstrates that kynurenine modulates macrophage behavior by directly inhibiting pyroptosis, a process that would otherwise restrict tumor growth through inflammatory cell death. Collectively, these findings suggest that kynurenine functions as a critical metabolic orchestrator in the TME, shaping immune landscapes to favor tumorigenesis.

Next, we investigated the molecular mechanism underlying kynurenine’s anti-pyroptotic effects in M2 macrophages and demonstrated that kynurenine directly interacts with CASP1, leading to reduced levels of CL-CASP1 in these cells. CASP1 functions as a central executor of pyroptosis, with its activation triggering the cleavage of gasdermin D (GSDMD) – the pore-forming protein essential for pyroptotic cell death [34]. By inhibiting CASP1 proteolytic processing, kynurenine effectively suppresses pyroptosis, thereby prolonging M2 macrophage survival. This mechanism aligns with emerging evidence that CASP1 activity is tightly modulated in the TME to regulate immune evasion and tumor progression. Our findings reveal that kynurenine-mediated inhibition of CASP1 cleavage constitutes a previously unrecognized mechanism through which tumors subvert the immune microenvironment to promote their own growth.

In addition, CASP1 overexpression in M2 macrophages abrogated the anti-pyroptotic effects of kynurenine and restored pyroptosis rates. This finding highlights the central regulatory role of CASP1 in mediating kynurenine-driven modulation of macrophage function. Furthermore, CASP1 overexpression neutralized kynurenine-induced enhancement of RCC cell proliferation, colony formation, and viability in co-culture systems. Collectively, these results suggest that targeting the kynurenine–CASP1 axis represents a promising therapeutic strategy for RCC.

This study has several limitations that warrant further investigation. The majority of experiments were primarily performed using *in vitro* co-culture systems, which may not fully replicate the physiological complexity of the TME *in vivo*. Future investigations should validate these findings using *in vivo* models, including orthotopic RCC mouse models and patient-derived xenografts, to better mimic clinical scenarios. Although we identified CASP1 as a critical mediator of kynurenine’s effects, the precise molecular mechanisms underlying its inhibition of CASP1 cleavage remain to be elucidated. Moreover, the clinical applicability of these findings requires further investigation in clinical settings to determine their translational potential.

In summary, our study reveals a previously unrecognized mechanism by which kynurenine directly binds to CASP1, suppressing its activation and subsequent pyroptosis in M2 macrophages. This finding is particularly significant because (1) it provides a direct link between tryptophan metabolism and pyroptotic cell death in macrophages, and (2) it explains how kynurenine-mediated immunosuppression in RCC may involve the inhibition of macrophage pyroptosis. Given that CASP1 is a central executor of pyroptosis, its inhibition by kynurenine represents a novel immune-evasion strategy in RCC, potentially contributing to the pro-tumorigenic effects of M2 macrophages. However, further research is needed to address the limitations of this study and to fully elucidate the clinical relevance of targeting the kynurenine–CASP1 axis in RCC.
